# Supplementary figures and images for: Bone marrow mesenchymal stem cell-derived exosomes protect podocytes from HBx-induced ferroptosis
Source: PeerJ. 2023 May 11;11:e15314. doi: 10.7717/peerj.15314 (PMC10183163; doi:10.7717/peerj.15314)

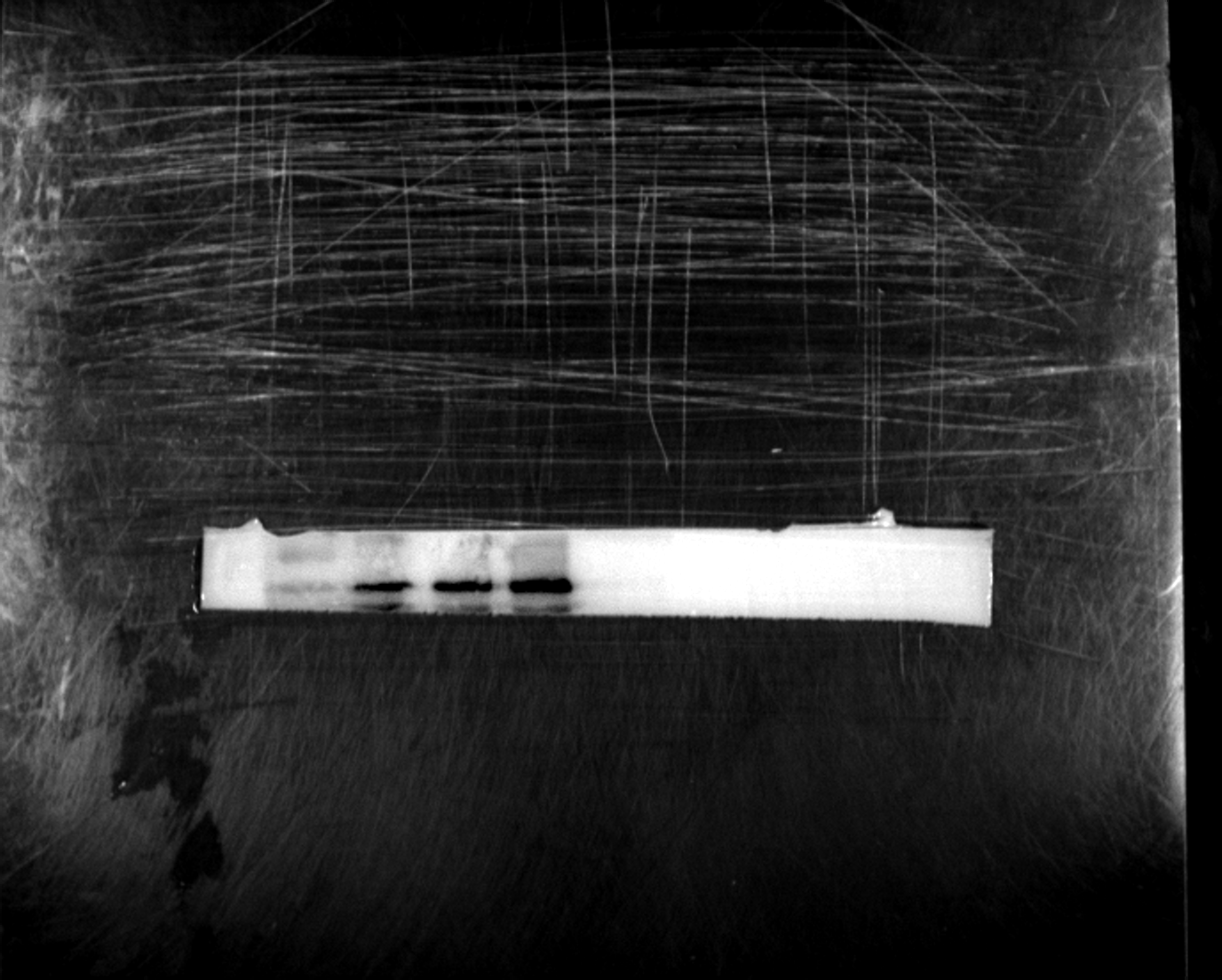

Supplement: Supplemental Information 2 [file peerj-11-15314-s002.zip › Part 1/ACSL4..Tif]

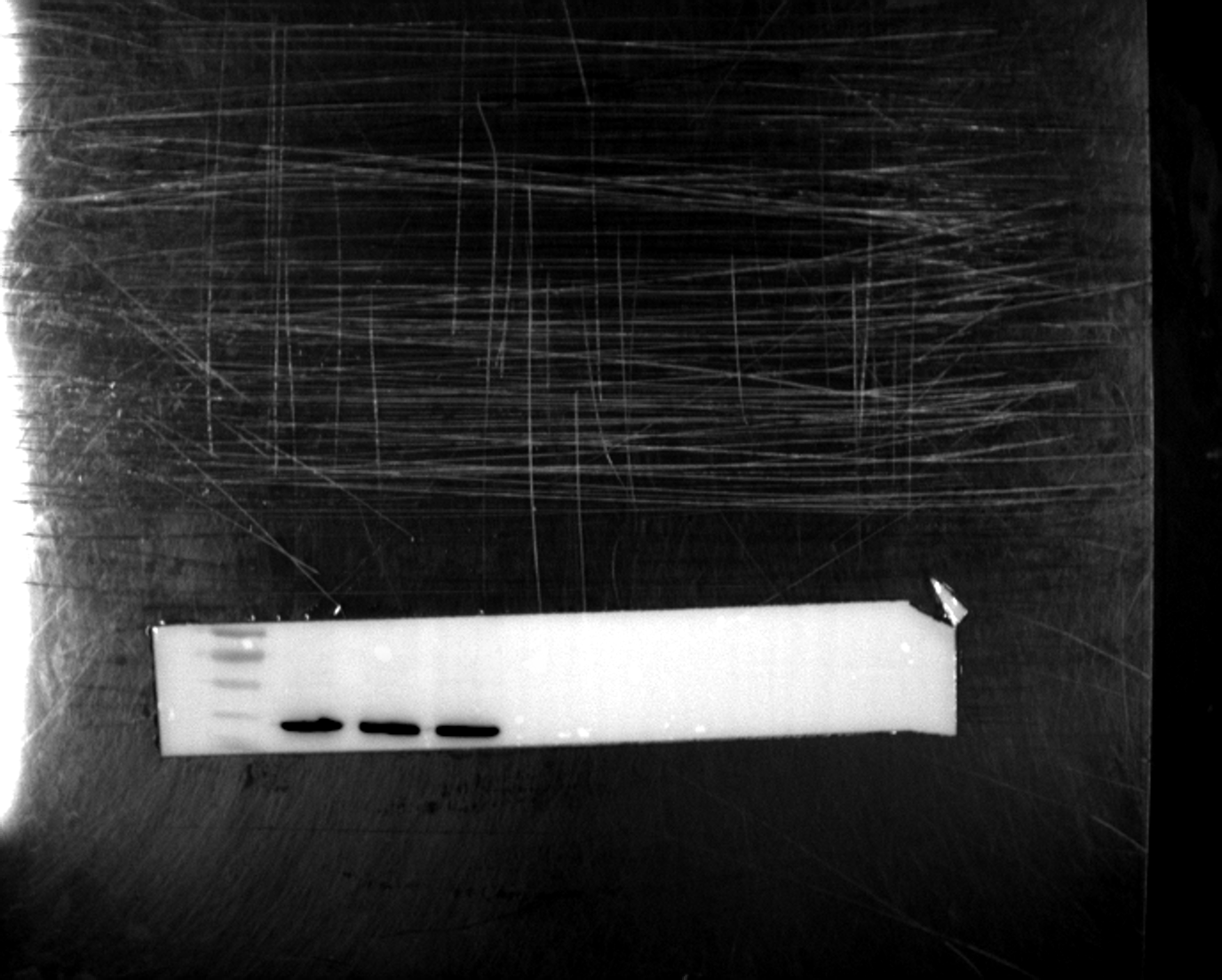

Supplement: Supplemental Information 2 [file peerj-11-15314-s002.zip › Part 1/GAPDH..Tif]

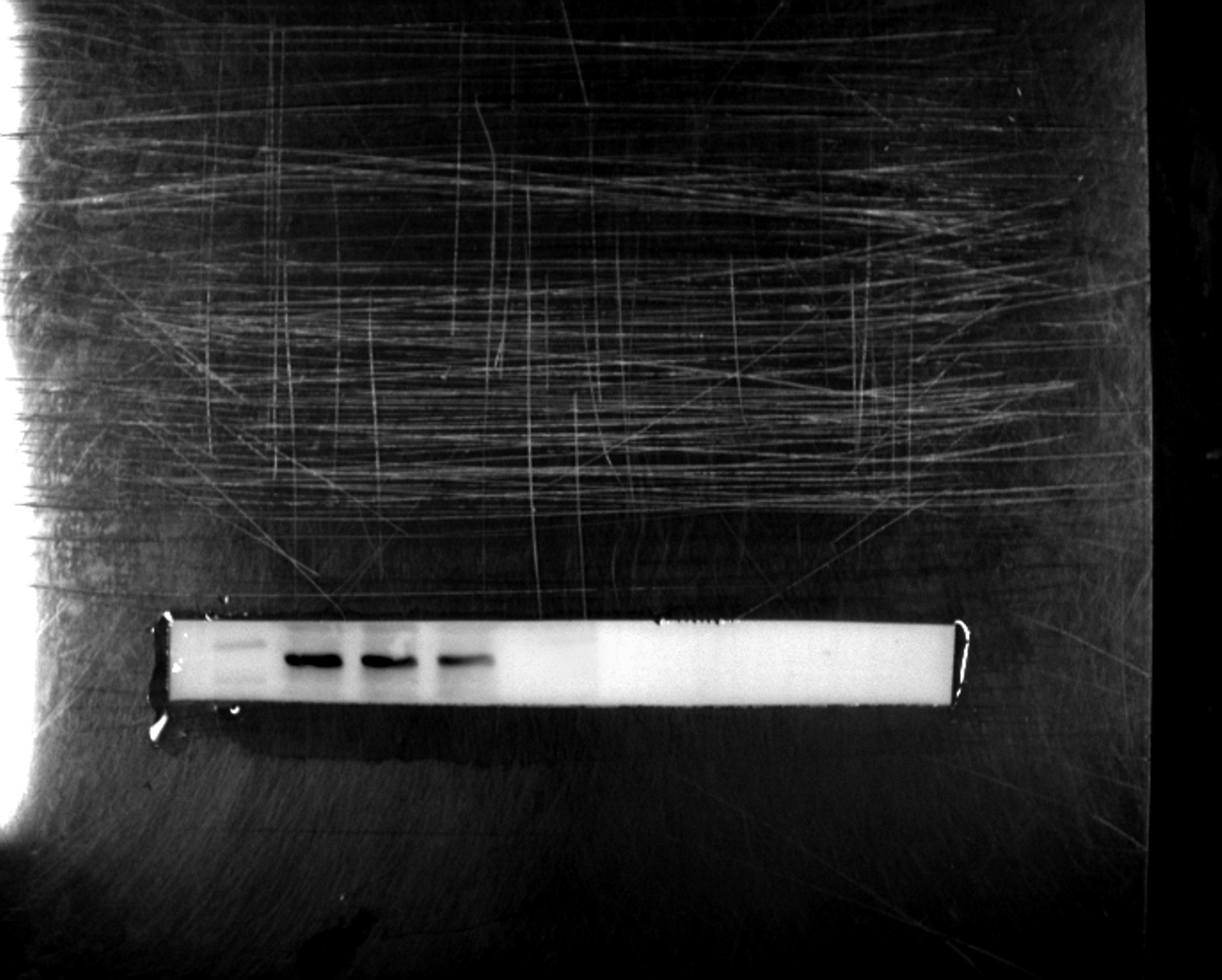

Supplement: Supplemental Information 2 [file peerj-11-15314-s002.zip › Part 1/GPX4..Tif]

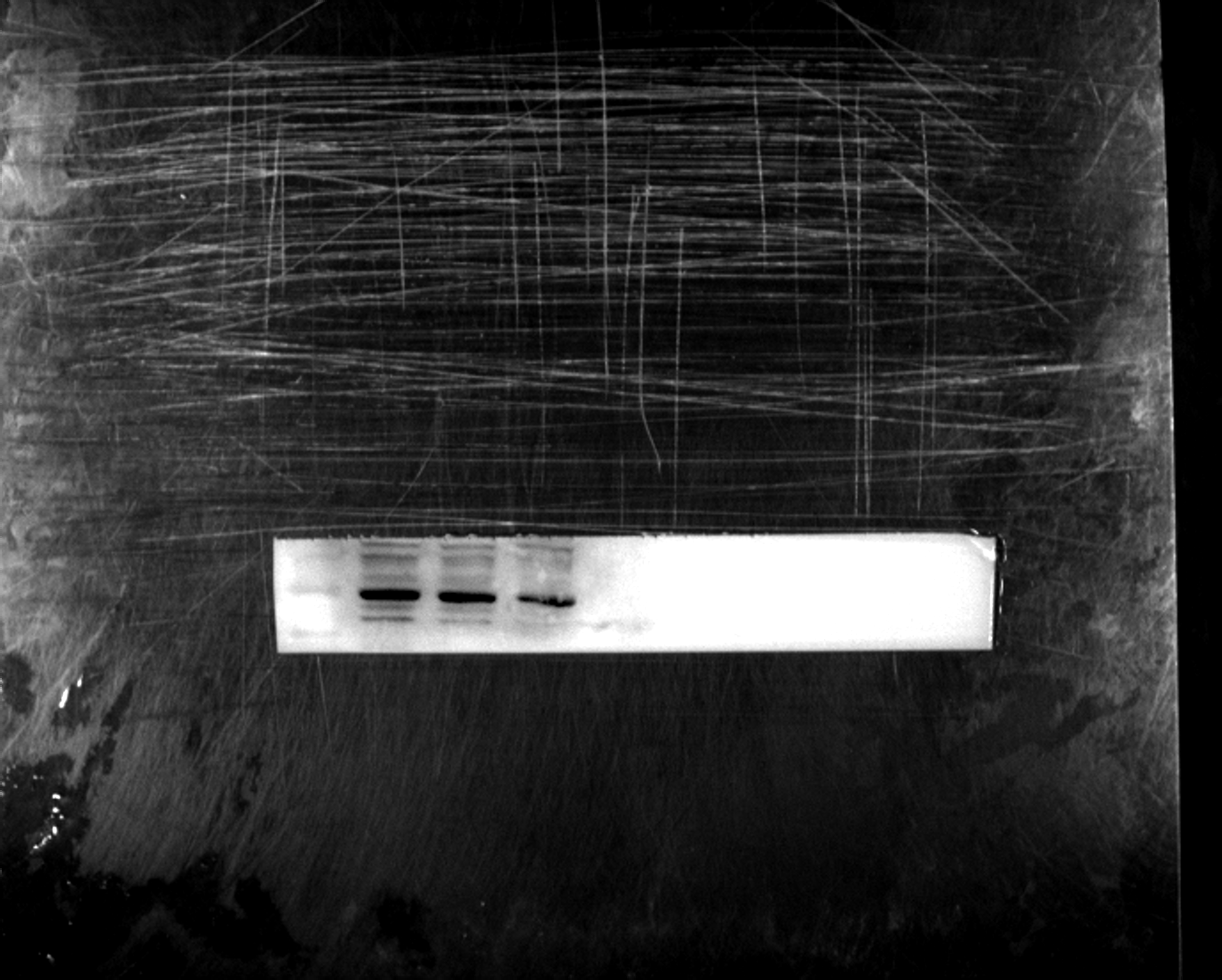

Supplement: Supplemental Information 2 [file peerj-11-15314-s002.zip › Part 1/SLC7A11..Tif]

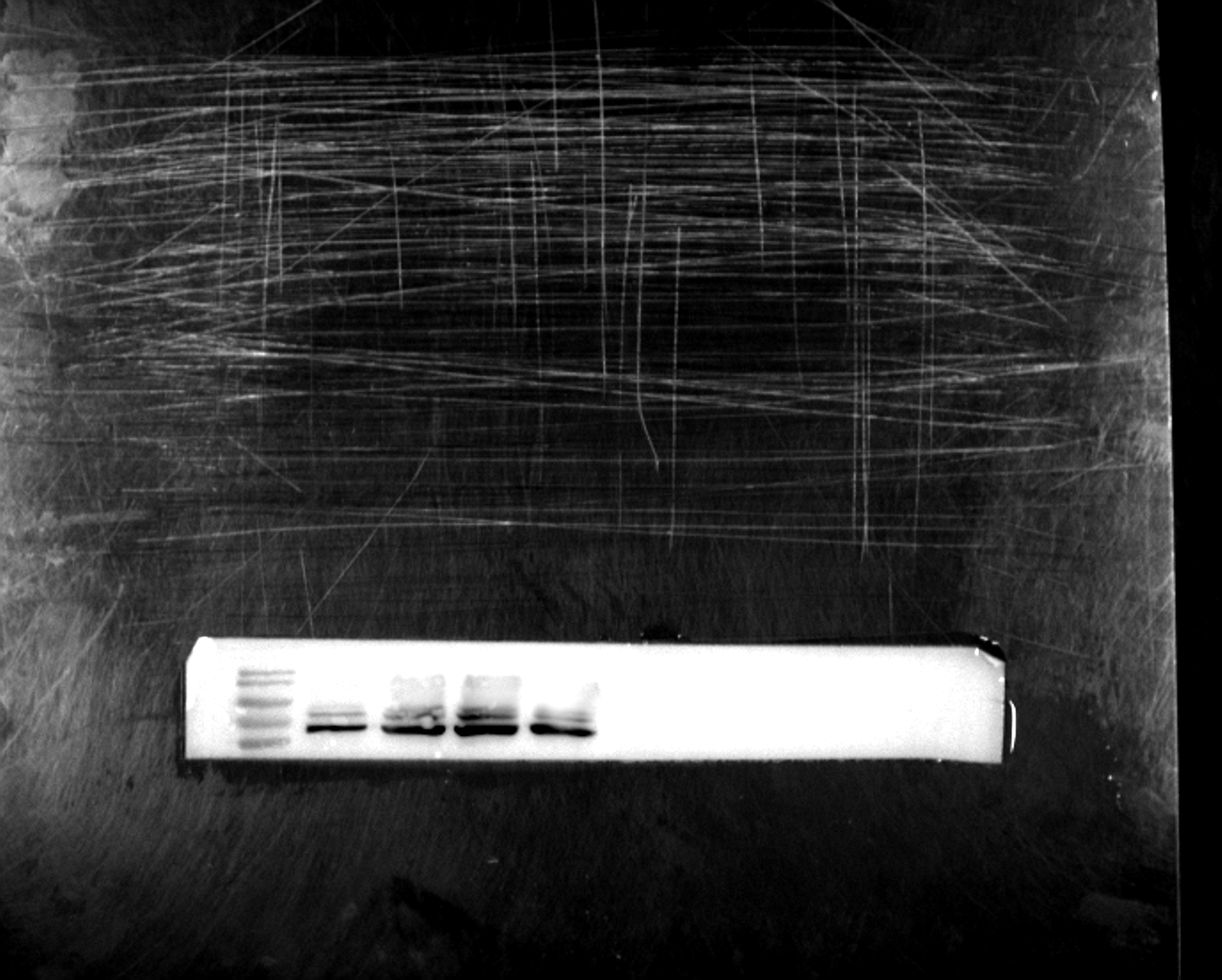

Supplement: Supplemental Information 2 [file peerj-11-15314-s002.zip › Part 3/ACSL4 ..Tif]

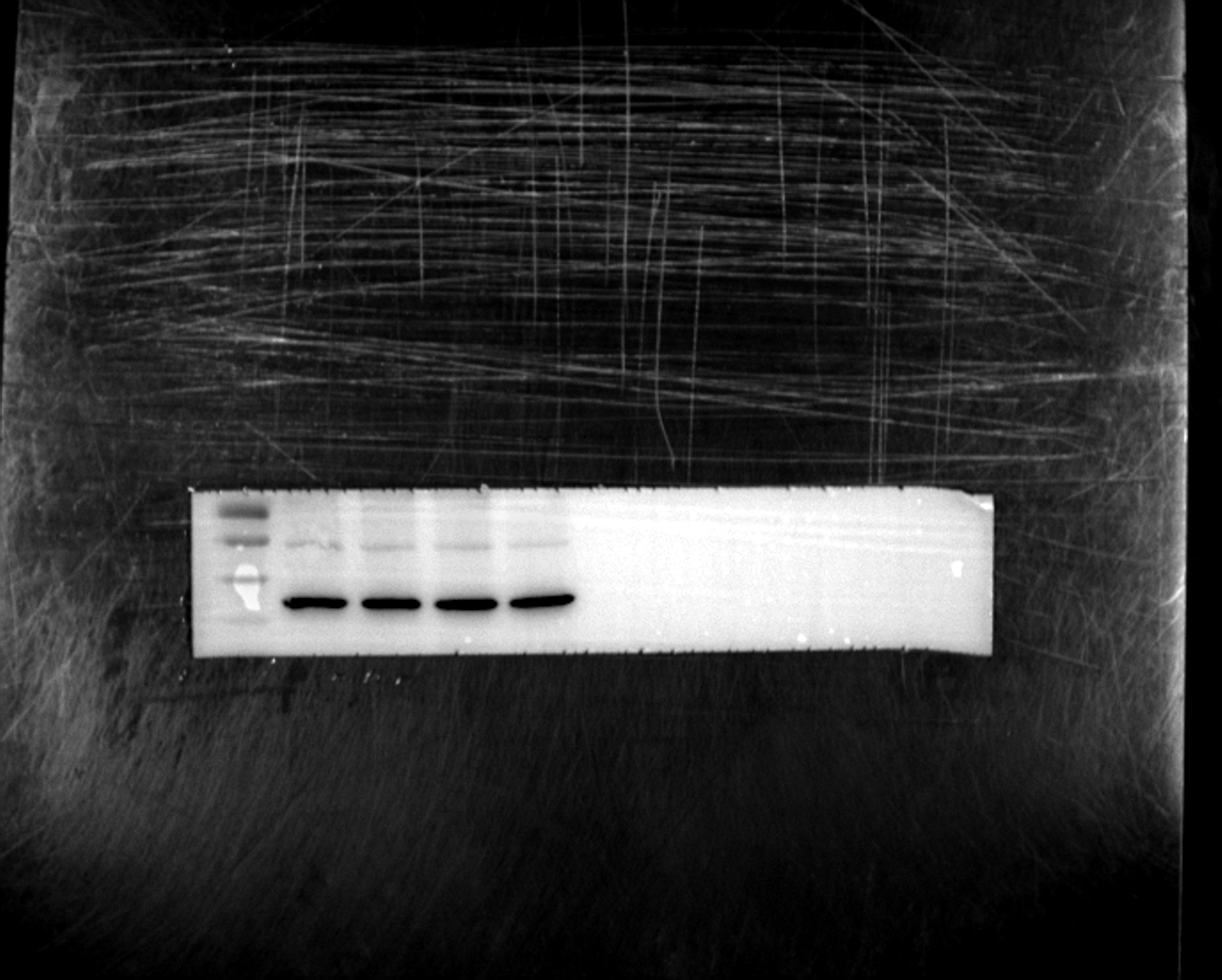

Supplement: Supplemental Information 2 [file peerj-11-15314-s002.zip › Part 3/GAPDH..Tif]

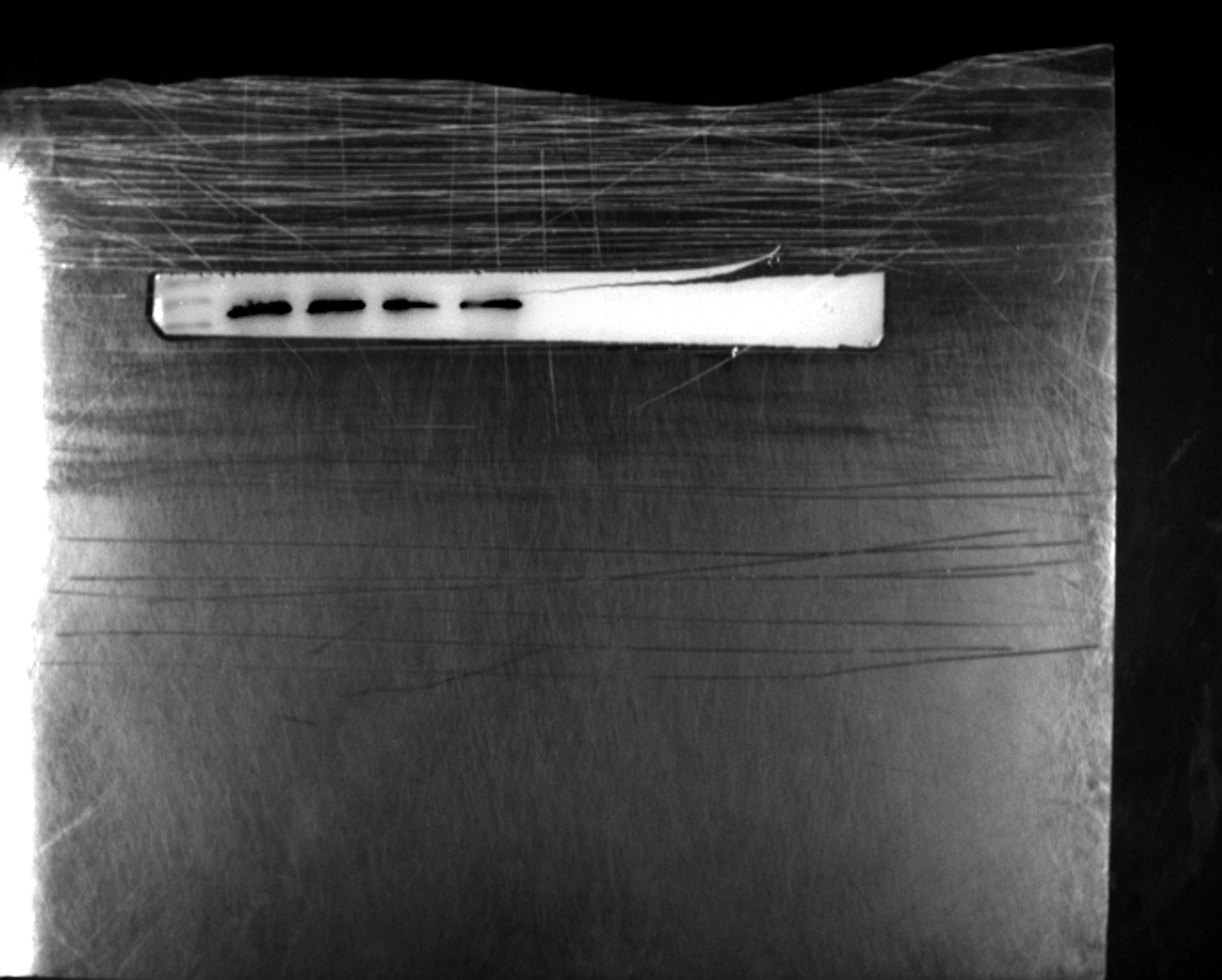

Supplement: Supplemental Information 2 [file peerj-11-15314-s002.zip › Part 3/GPX4 ..Tif]

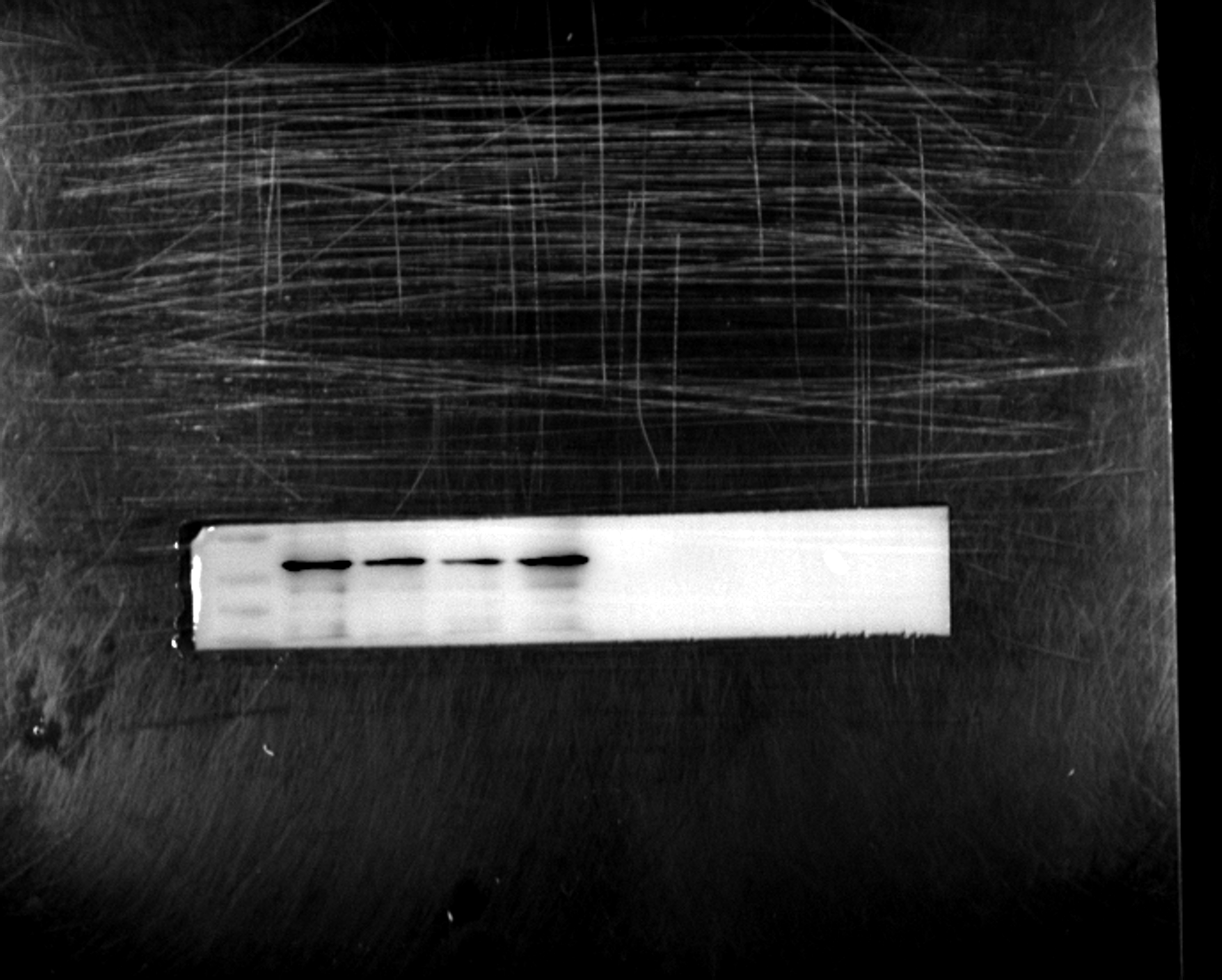

Supplement: Supplemental Information 2 [file peerj-11-15314-s002.zip › Part 3/SCL7A11..Tif]

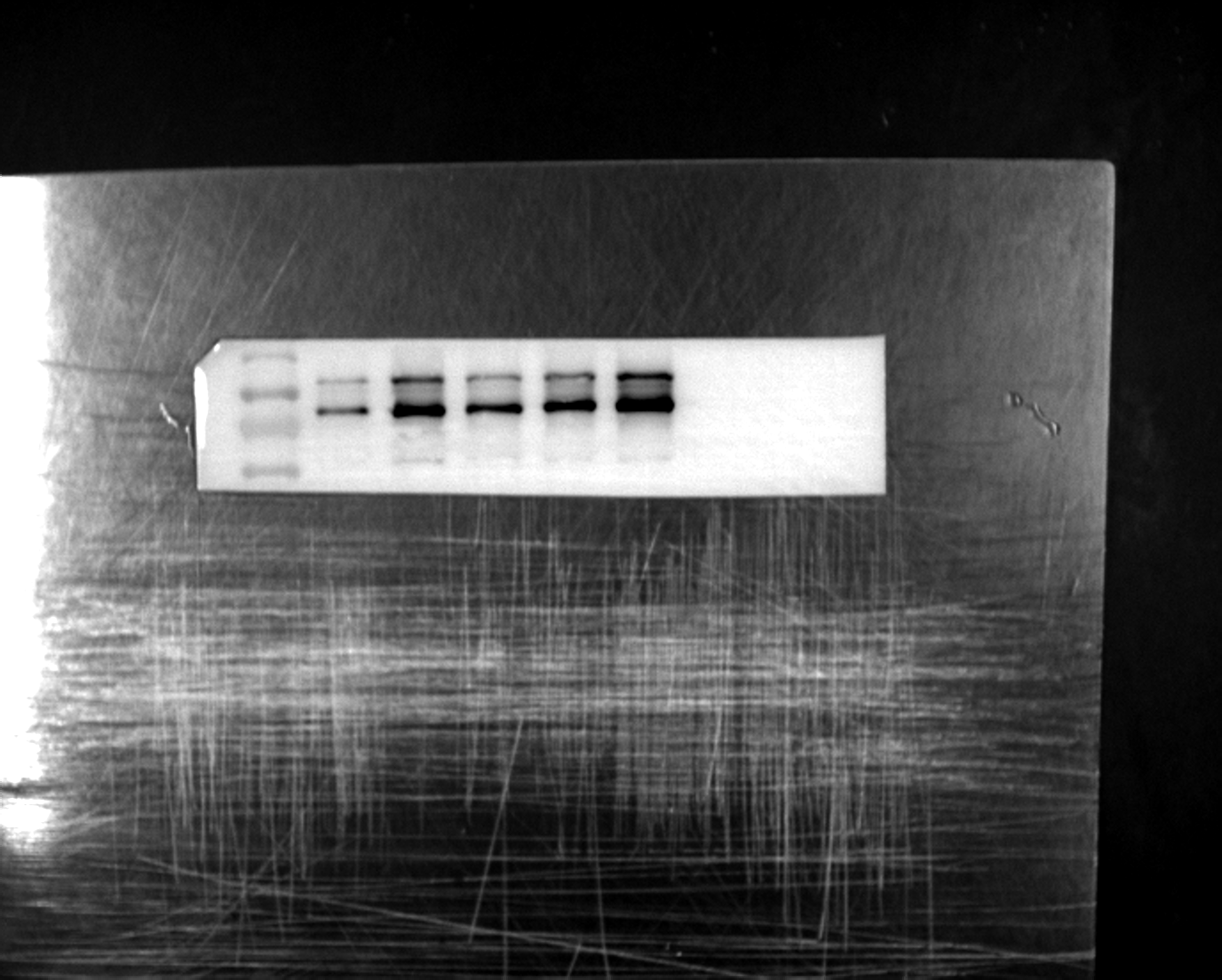

Supplement: Supplemental Information 2 [file peerj-11-15314-s002.zip › Part 4/ACSL4..Tif]

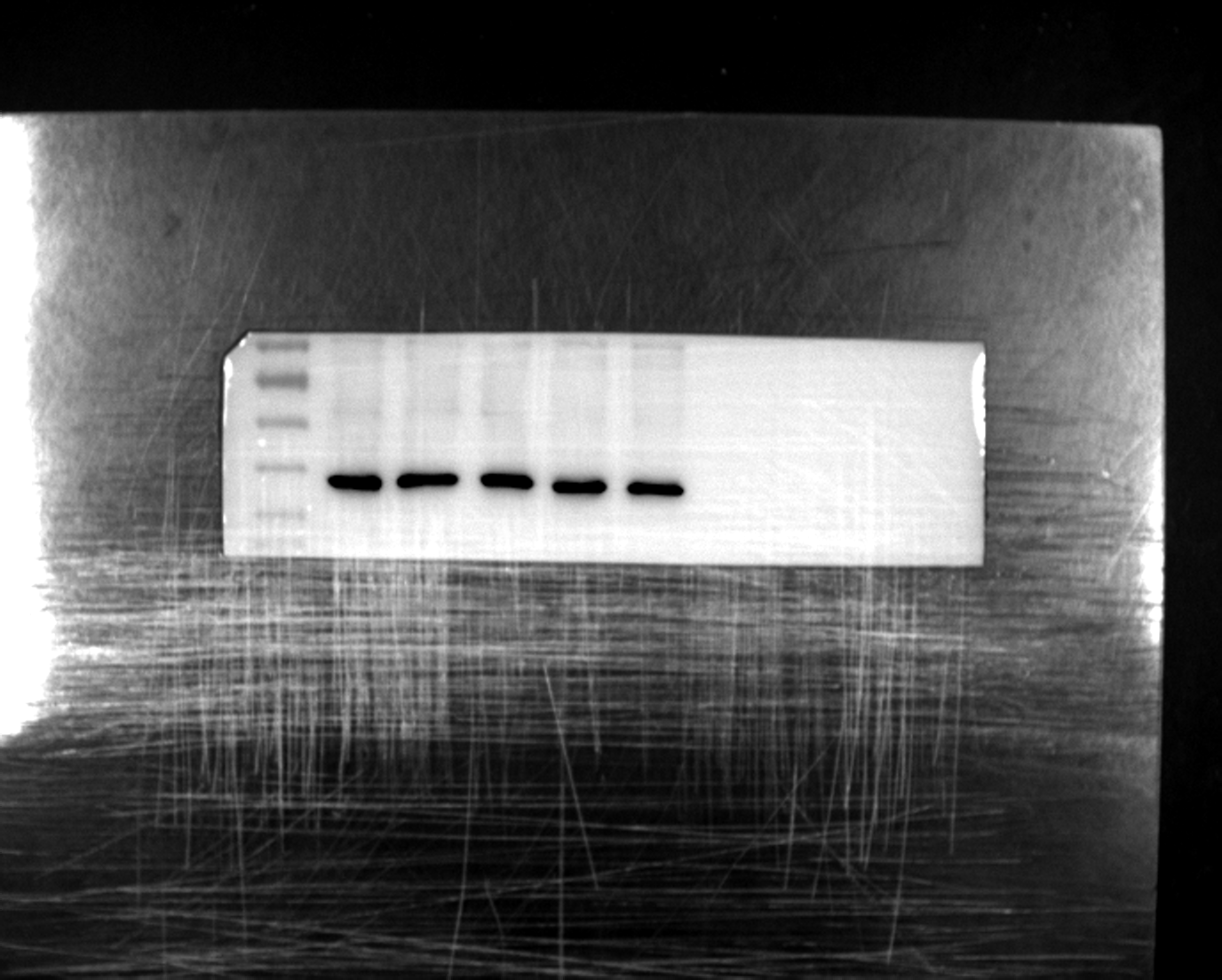

Supplement: Supplemental Information 2 [file peerj-11-15314-s002.zip › Part 4/GAPDH..Tif]

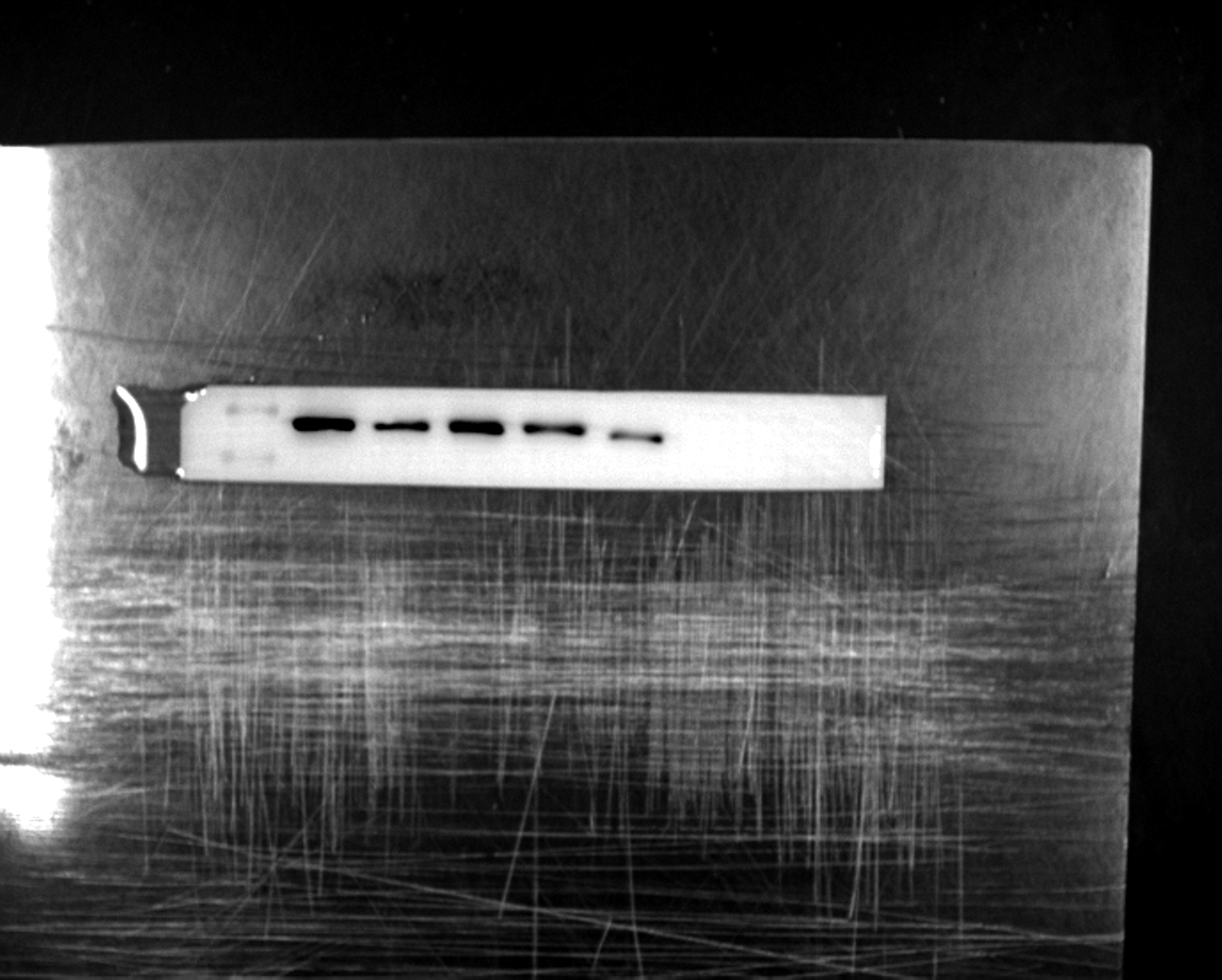

Supplement: Supplemental Information 2 [file peerj-11-15314-s002.zip › Part 4/GPX4..Tif]

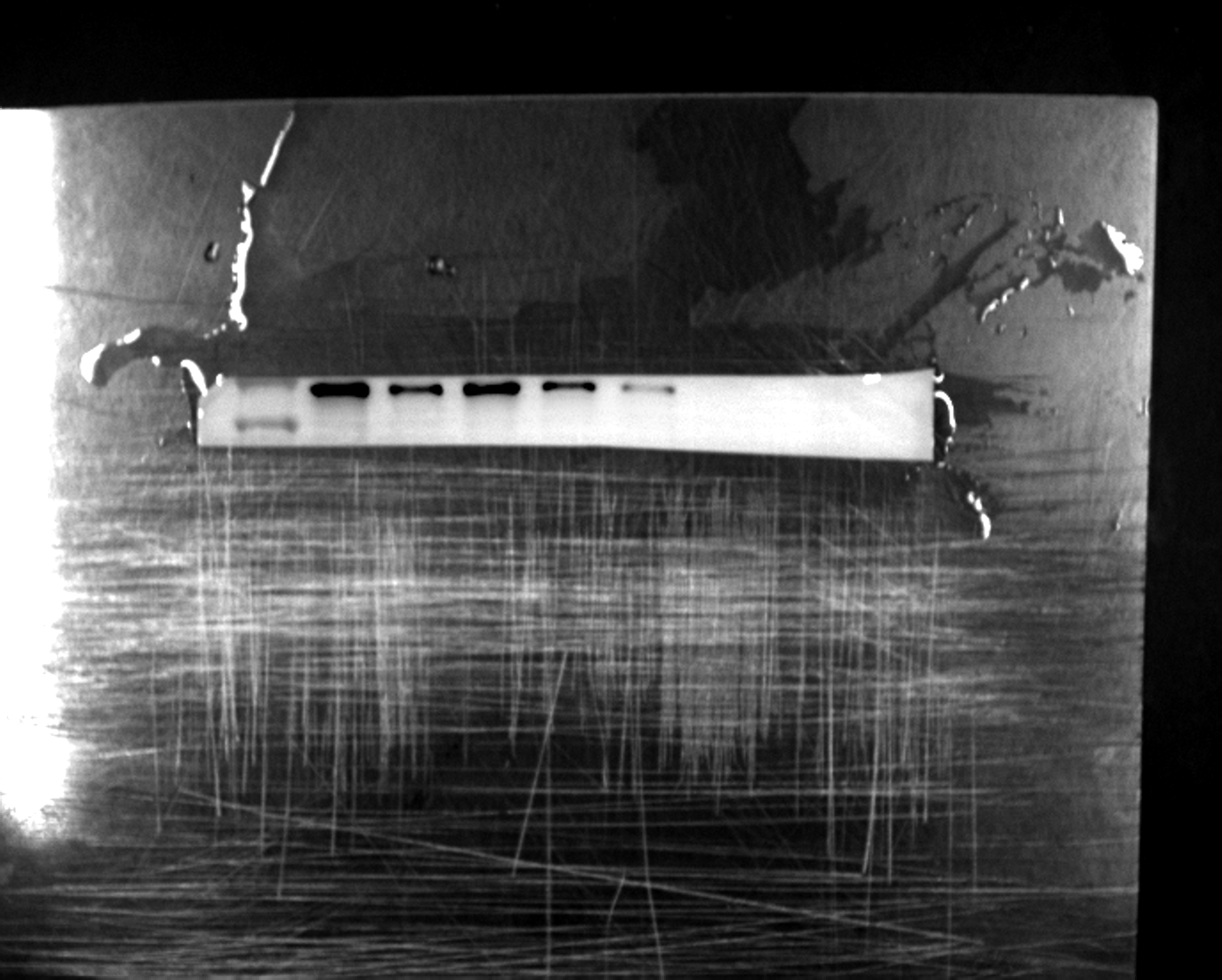

Supplement: Supplemental Information 2 [file peerj-11-15314-s002.zip › Part 4/SLC7A11..Tif]
